# Rural medical workforce pathways: exploring the importance of postgraduation rural training time

**DOI:** 10.1186/s12960-023-00819-3

**Published:** 2023-04-20

**Authors:** Matthew R. McGrail, Tiana Gurney, Jordan Fox, Priya Martin, Diann Eley, Bushra Nasir, Srinivas Kondalsamy-Chennakesavan

**Affiliations:** 1 Rural Clinical School, The University of Queensland, Rockhampton, QLD 4700 Australia; 2 Rural Clinical School, The University of Queensland, Toowoomba, QLD 4350 Australia; 3 Academy for Medical Education, The University of Queensland, Herston, QLD 4006 Australia

**Keywords:** Rural workforce, Medical education, Training pathways, Distribution, Rural origin, Doctors, Postgraduation training, Graduate medical education, General practice

## Abstract

**Background:**

Inadequate distribution of the medical workforce in rural regions remains a key global challenge. Evidence of the importance of postgraduation (after medical school) rural immersion time and subsequent rural practice, particularly after accounting for other key factors, remains limited. This study investigated the combined impact of three key training pathway factors: (1) rural background, (2) medical school rural immersion, and (3) postgraduation rural immersion, and duration time of each immersion factor on working rurally.

**Methods:**

Data from a cross-sectional national survey and a single university survey of Australian doctors who graduated between 2000 to 2018, were utilised. Key pathway factors were similarly measured. Postgraduation rural training time was both broad (first 10 years after medical school, national study) and specific (prevocational period, single university). This was firstly tested as the dependent variable (stage 1), then matched against rural practice (stage 2) amongst consultant doctors (national study, *n* = 1651) or vocational training doctors with consultants (single university, *n* = 478).

**Results:**

Stage 1 modelling found rural background, > 1 year medical school rural training, being rural bonded, male and later choosing general practice were associated with spending a higher proportion (> 40%) of their postgraduation training time in a rural location. Stage 2 modelling revealed the dominant impact of postgraduation rural time on subsequent rural work for both General Practitioners (GPs) (OR 45, 95% CI 24 to 84) and other specialists (OR 11, 95% CI 5–22) based on the national dataset. Similar trends for both GPs (OR 3.8, 95% CI 1.6–9.1) and other specialists (OR 2.8, 95% CI 1.3–6.4) were observed based on prevocational time only (single university).

**Conclusions:**

This study provides new evidence of the importance of postgraduation rural training time on subsequent rural practice, after accounting for key factors across the entire training pathway. It highlights that developing rural doctors aligns with two distinct career periods; stage 1—up to completing medical school; stage 2—after medical school. This evidence supports the need for strengthened rural training pathways after medical school, given its strong association with longer-term decisions to work rurally.

## Introduction

Globally, adequate distribution of the medical workforce into regional, rural and remote communities continues to be a major challenge of many countries [1, 2], despite longstanding goals of good health for all and reduced within-country inequities [3]. Several recent reviews highlight growing evidence of the effectiveness of available solutions [4–7], with increased selection of rural origin students and more distributed training of students in rural areas being commonly associated with increased recruitment and retention of rural doctors. Broadly, evidence of rural workforce retention relates to at least one of the World Health Organization’s identified four domains of; financial incentives, education and training, regulatory strategies and supports for rural practice [8, 9].

The domain of education and training rightly attracts much of the policy focus and evidence to date, given that long-term growth of the rural medical workforce is reliant on sufficient uptake from the next generation of doctors. The majority of evidence around rural medical workforce distribution relates to increased selection of students with a rural background and increased periods of rural immersion within medical school training [10–13]. In contrast, few studies have reported on the impact of postgraduation (i.e. after medical school) rural training on rural medical workforce distribution [14–17]. It is important to understand all periods, as training a doctor to the point of independent practice is a long pathway across all medical school (including pre-medicine) and postgraduation qualifications (prevocational periods in some contexts and registrar training enrolled with a specialty college), as well as their time prior to university (childhood).

Within each stage of their pathway an individual may develop an interest or preference to a specific location or type of location, that they eventually choose to work and/or reside in. The potential influence of each stage (i.e. the observed intervention) on increasing the rural workforce supply is generally assessed by measuring either the length of training time spent rurally, or as a binary factor meeting a minimum ‘exposure’ definition (for example, being of rural background if they lived more than 5 years of their childhood in rural locations). Multiple studies have confirmed the strong association between having a rural background and later working rurally as a doctor [18, 19]. Similarly, multiple studies have demonstrated a significant association between medical school rural training immersion and subsequent rural work, even after adjustment for rural background [11, 20, 21]. However, few studies have focused on the association between postgraduation rural training time and subsequent rural work, particularly when considered in combination with other factors of the pathway.

Evidence suggests that a doctor’s decision to work rurally, or not, develops over time across each factor of their pathway but is commonly dependent on their choice of specialty [22, 23]. General practice is strongly amenable with practising rurally in most locations, whilst other key generalist specialties (e.g., internal adult medicine, general surgery, paediatrics, obstetrics) are sustainable in most larger rural towns [24, 25]. Moreover, many rural-based general practice vocational (or residency) training programmes openly prepare such graduates specifically for rural practice [26, 27]. In contrast, many specialties (including sub-specialists) dictate them working in either a large metropolitan or regional-level hospital, or through an outreach or other visiting service model. Other influences known to potentially modify or moderate a doctor’s willingness or interest in working rurally include having a life partner, their partner’s profession and childhood background, having dependents and their schooling needs, as well as specific hobbies and interests [9, 28–30]. Measuring the impact of these factors on a doctor’s practice location decision has rarely been quantified [31], particularly when in consideration of training pathways [12].

### Context of this study

Australia provides a useful setting for studying the association between training pathways of the medical workforce and distribution of the workforce, given its long-term national policies supporting rural medical training and its large geography of dispersed populations. Formal rural training programmes largely gained momentum in the 1990s, with key developments being the expansion of medical school places and medical schools located outside of capital cities, the establishment of the Rural Clinical Schools program in 2000 and subsequent requirements for half of general practice training to occur outside of metropolitan areas. More recently, there has been an expansion of wholly rural (end-to-end) medical school pathways whilst the Regional Training Hubs program from 2017 has increased the spotlight on both expanding and strengthening rural training pathways beyond medical school for all specialties. In 2021, Australia also published its 10-year National Medical Workforce Strategy, with a key focus on addressing the ongoing geographic maldistribution [2].

This study aimed to explore the combined impact of three key training pathway factors (rural background, medical school rural immersion, postgraduation rural immersion) and duration time of each immersion type on working rurally amongst Australian-trained doctors. It draws upon two complementary datasets, each of which contain different attributes and measures of medical school and postgraduation rural training immersions, to describe the various pathways of individual doctors and how they relate to developing a rural medical workforce. Table 1 provides an overview of Australia’s career stage pathway for most doctors, from before medical school to completion of all training, along with an approximate timeframe for each stage of the pathway.

**Table 1:** Summary of Australia’s medical training pathway for individual doctors

Training period	Career stage	Description	Time (years)
Schooling	Childhood	Period(s) of their upbringing (childhood) that may have been in a rural setting	Up to 18 years
University qualification(s)	Pre-medicine	Initial degree (e.g., Medical Science), prerequisite for a majority of medical degrees	Usually 3 years
	Medical school	Initial medical degree (e.g., Doctor of Medicine); some pathways integrate both qualifications (including pre-medicine) as one (5–6 years); latter half of medical school is predominantly in clinical settings	Usually 4 years
Postgraduation qualifications	Prevocational	Compulsory intern year; then additional 2–3 years (on average) working in a hospital either rotating between different departments or in unaccredited training positions; Employment is frequently via an annual contract, with most such doctors actively seeking selection into specialty training. This is when most specialty decisions are confirmed (with future work location strongly associated with this decision)	Minimum 2 years, median 3–4 years
	Vocational (registrar)	Doctor works as a registrar in accredited training positions that meet specialty college requirements. Employment is frequently via an annual contract, dependent on supervision, accreditation, and specific learning needs	3–6 years
Independent	Consultant	Largely unrestricted period of working as a specialist doctor	n/a

## Methods

This retrospective cohort study design used data from two sources, namely (1) the Medicine in Australia: Balancing Employment and Life (MABEL) study, a large longitudinal (annual cohort) survey of the Australian medical workforce and (2) the University of Queensland’s Medical graduates Cohort Study (UQMediCoS), a cross-sectional survey of its medical graduates. The MABEL study invited all identified doctors in Australia to complete its initial survey in 2008, with respondents forming a ‘panel’ who then received an annual invite to complete an updated survey through to 2018 [32]. Each subsequent year there was an annual top-up of invitees sourced from new doctors in Australia. Most years the panel received both a hardcopy survey and a link to the online version, with three reminders. The UQMediCoS study most recently invited all University of Queensland (UQ) medical school graduates of 2002–2018, excluding international students and those not registered with the Australian Health Practitioner Regulation Agency, to complete a survey in 2019 about their training and career decisions. Invites were distributed via their last known email address, with two reminders. MABEL was approved by the University of Melbourne Faculty of Economics and Commerce Human Ethics Advisory Committee (Ref. 0709559), whilst UQMediCoS was approved by The University of Queensland Human Research Ethics Committee (Ref. 2018001630 and 2012001171).

Both datasets are described below:

- MABEL (2018) provides representative national-scale data, free of jurisdictional or single-university bias. It contains a large number of observations with low counts of missing data. However, training related data were not its primary focus with measures needing to be generic to suit all graduates. As such, rural background does not wholly align with the government’s definition, medical school rural training time categories are generalised to fit all programmes nationally, whilst postgraduation rural training time cannot be separated by career stages; in particular, prevocational and registrar periods. The latter measure was only asked in the final year (2018) of the MABEL survey.
- UQMediCoS (2019) provides single university outcomes data with definitions of rural background and medical school rural training time aligned with national policy, as well as postgraduation rural training time split across the prevocational and registrar periods. However, its response numbers are substantially less than MABEL, there is a higher proportion with missing data, and workforce outcomes are biased to one jurisdiction.

The primary rationale for parallel analyses of the MABEL and UQMediCoS datasets is that individually each has important strengths (but also limitations), thus combining their results provides some validation of each against the other, thus strengthening the overall presented evidence. Differences in the definitions between the key study measures in each dataset mean that it is valuable to examine these results in tandem. As outlined below, the data for each study were collected at a similar timepoint, with some exclusion criteria making the set of participants highly comparable (see explanation notes in Table 2). Additionally, the range of key variables collected across both datasets was very similar.

**Table 2:** Definitions, inclusion criteria and differences of data items drawn from the UQMediCoS and MABEL datasets

Factor	UQMediCoS	MABEL	Explanation (differences)
University	University of Queensland	National (all Australian universities)	Aggregate of ~ 20 programmes vs one programme
Cohort	Graduates 2002–2018	Graduates 2000–2017	Excludes < 2000 for MABEL
Response year	2019 (1–17 years postgraduation)	2018 (1–18 years postgraduation)	Similar periods
Exclusion	International students, those not practising	International students, those not practising	Same
Rural background	6 consecutive years rural or 10 aggregate years	At least 6 aggregate years rural	MABEL uses an approximation only of the policy definition
MS rural time	3 options: 0, 1 or 2 years	3 options: < 12 weeks, 3–12 months; > 1 university year	Categories are similar, but not equivalent
PG rural time	Continuous proportion (0–100% of prevocational period)	Discrete values: 0 to 10 years (across prevocational and vocational period)	As noted, relate to different career stages
Career stage	Combined observed outcome for registrars and consultants	Outcome for consultants only	Differences largely due to rural P/G time definitions
Rural bonded	Those awarded practise restricting scholarships or bonded places	Those subject to restrictions on where they practise	Similar, but not equivalent
Gender	Male, female	Male, female	Same
Age	At graduation < > 28 +	At graduation < > 28 +	Same
Specialty	General practice, any other specialist	General practice, any other specialist	Similar (UQMediCoS includes registrars)

*MS rural time* medical school rural (clinical) training time, *PG rural time* postgraduation rural training time

### Study measures and definitions

Most measures relevant to this study from the MABEL and UQMediCoS datasets used either a slightly different method or applied to a slightly different context, when compared to each other. All differences between the two datasets are summarised in Table 2. The largest divergences between the two datasets relate to the measurement of rural training times. Firstly, medical school rural training time has somewhat similar categories, but they are not directly comparable. This is due to MABEL using categories suitable for the breadth of programmes nationally (options were < 12 weeks, 3–12 months and > 1 year), whereas UQMediCoS categories were specifically aligned with that organisation’s programme (options were 0, 1 or 2 years). Secondly, postgraduation rural training time was collected using somewhat different approaches. MABEL asked “*During the first 10 years after completing your basic medical degree, how many years (0 to 10) did you spend training or working in a rural area?”;* UQMediCoS asked “*How many months/years did you spend training/working in each location type (categories of the Modified Monash Model, MMM rurality classification *[33])* after graduating in three discrete periods (prevocational training; during registrar training; after registrar training)?”.*

UQMediCoS data relate to graduates between 2002–2018 and were collected in 2019, thus respondents graduated between 1 and 17 years ago. MABEL data were collected in 2018, but graduation year of respondents ranged from 1 to 60 years prior to 2017. For this study, MABEL participants who graduated prior to 2000 were excluded to warrant comparability. Furthermore, doctors who are still in their prevocational training stage were excluded from both datasets for two reasons. Firstly, a key objective of this study is to measure the impact of postgraduation rural training time on subsequent work locations, but such analysis of data from prevocational doctors is problematic given that these doctors are observed still within this period. Secondly, given the expected strong relationship between general practice and rural training pathways, analyses are stratified by specialty (general practitioner (GP), other), thus excluding those without a confirmed specialty (prevocational).

MABEL’s postgraduation rural training time measure prevented separation by career stages. For this study MABEL data were assumed to approximate the combined prevocational and vocational training periods (i.e. during the first 10 years), thus only doctors who had completed both stages were included in analyses. In contrast, the UQMediCoS study separated postgraduation rural training time by career stages. Its smaller participation counts limited modelling to include both doctors still completing vocational training and those who had completed all training.

### Key outcomes and comparison groups

Postgraduation rural training time, used as both an outcome and independent variable, was converted to a percentage and grouped in 10% values, enabling visual comparison between the two datasets. For most analyses, these were aggregated into three groups, defined as 0–10% (‘minimal’), 11–40% (‘some’) and 41–100% (‘frequent’). Work location was self-reported as town/suburb and postcode, then geocoded under the MMM national classification as rural for MMM 2–7 communities or metropolitan for MMM-1.

### Statistical analysis

For each dataset, multivariable regression models were applied to two separate outcomes; firstly (stage 1), the proportion of postgraduation training time they spent in a rural location applied a multinomial model; secondly (stage 2), currently working rurally used a logistic model. Each of these models included all variables as defined in Table 2, with postgraduation rural training time changing from the dependent variable in the first model to an independent variable for the second model. Specialty (observed as a confirmed decision later in their pathway) was applied as an independent variable to the stage 1 model and used to stratify results in the stage 2 model. Following these, a set of pathways associated with working rurally was described using MABEL and UQMediCoS data by combining the three key factors of rural background, medical school rural training time and postgraduation rural training time, stratified by specialty. All analyses used Stata SE 15.1 for Windows (Stata Corp, College Station, TX, USA) and *p* < 0.05 for statistical significance.

## Results

There were 1651 (MABEL) and 478 (UQMediCoS) responses meeting the inclusion criteria (Table 2), with 28.1% and 24.9%, respectively, working rurally (Table 3). One key difference of study characteristics was that GPs were 48.6% of MABEL’s dataset compared with 36.4% from UQMediCoS, largely due to the exclusion of MABEL’s registrars (who are more likely to be other specialists, due to their longer training pathway). Medical school rural training times were similarly distributed, with males and aged 28 + at graduation more common in the UQMediCoS dataset. Postgraduation rural training time proportions were similar, though the > 40% rural training category was more commonly observed in UQMediCoS, likely because this study’s measurement related only to the prevocational training period. Apart from gender, all factors were related to increased proportions working rurally in both datasets. The largest discrepancy was with postgraduation rural training time having a clearer dose effect with working rurally in the MABEL dataset (i.e. each category increase in postgraduation rural time saw a similar proportional increase of those working rurally), partly because this study’s measurement included both prevocational and vocational periods and the outcome related to when doctors are fully trained and thus more independent in their choice of work location.

**Table 3:** Characteristics of included study participants and their association with working rurally

Factor	Groups	MABEL—National graduates		UQMediCoS—UQ only graduates	
		*N* (%)	Work rural (%)	*N* (%)	Work rural (%)
		*N* = 1651	457 (28.1%)	*N* = 478	116 (24.9%)
Specialty	GP	802 (48.6%)	310 (39.5%)	174 (36.4%)	67 (39.0%)
	Any other	849 (51.4%)	147 (17.4%)	304 (63.6%)	49 (16.7%)
Rural BG	Yes	386 (27.0%)	174 (45.2%)	117 (26.2%)	44 (38.3%)
	No	1044 (73.0%)	219 (21.2%)	330 (73.8%)	63 (19.6%)
MS rural training	0 years	XX	XX	328(68.6%)	58 (18.1%)
	1 year	XX	XX	89 (18.6%)	34 (39.1%)
	2 years	XX	XX	61 (12.8%)	24 (40.7%)
	Nil to 12 weeks	1181 (71.5%)	300 (25.9%)	XX	XX
	3–12 months	352 (21.3%)	102 (29.1%)	XX	XX
	> 1 year	118 (7.2%)	55 (46.6%)	XX	XX
PG rural training	Nil to 10% rural	841 (56.6%)	87 (10.4%)	250 (53.9%)	41 (16.9%)
	> 10 to 40% rural	371 (25.0%)	118 (32.1%)	89 (19.2%)	13 (15.3%)
	> 40% rural	275 (18.5%)	214 (78.4%)	125 (26.9%)	59 (47.6%)
Gender	Male	672 (40.7%)	184 (27.7%)	227 (47.5%)	61 (27.5%)
	Female	979 (59.3%)	273 (28.3%)	251 (52.5%)	55 (22.5%)
Rural bonded	Yes	150 (9.1%)	88 (59.5%)	62 (13.0%)	29 (47.5%)
	No	1501 (90.9%)	369 (24.9%)	416 (87.0%)	87 (21.5%)
Age 28 + (grad)	Yes	400 (25.9%)	134 (34.0%)	145 (30.3%)	48 (34.3%)
	No	1146 (74.1%)	277 (24.4%)	333 (69.7%)	68 (20.9%)

*Rural BG* rural background, *MS rural training* medical school rural (clinical) training time, *PG rural training* postgraduation rural training time, *GP* general practitioner

**Fig. 1:**
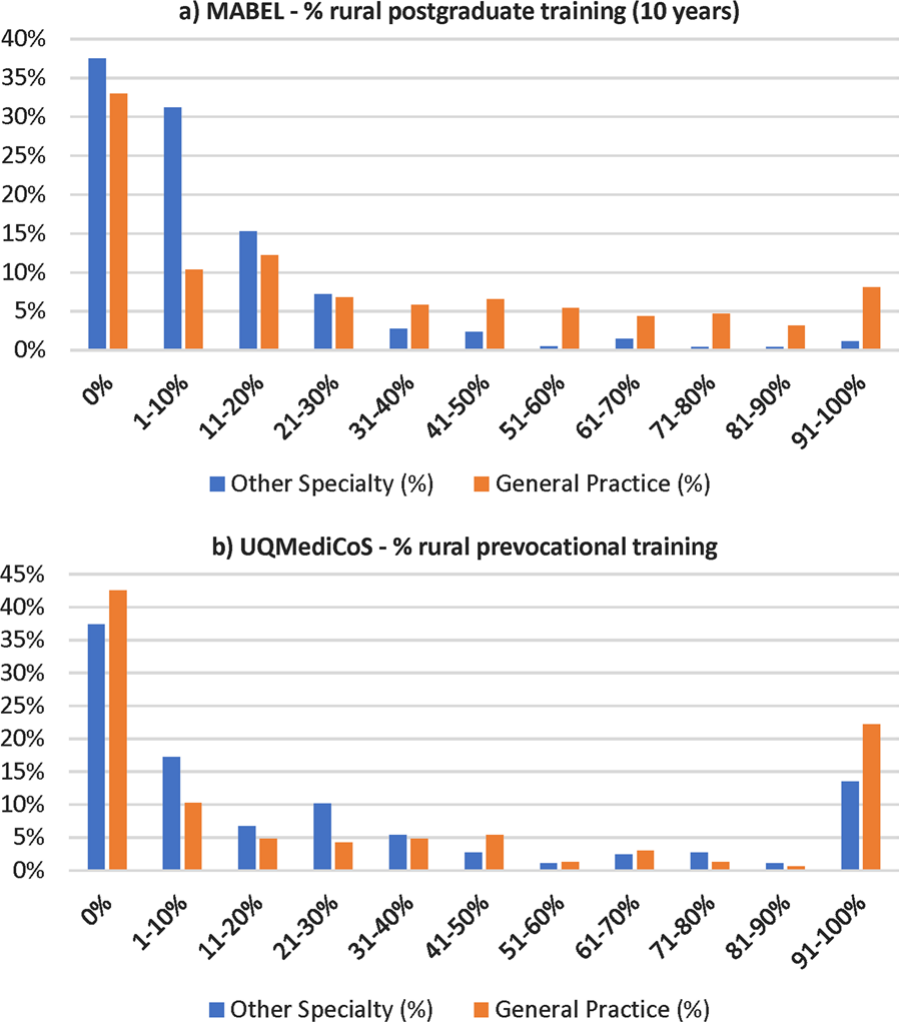
Proportion of postgraduation rural training time in the MABEL^1^ and UQMediCoS^2^ datasets. ^1^MABEL—consultant doctors only, rural time relates to first 10 years after basic medical degree. ^2^UQMediCoS—registrars and consultants, rural time relates to prevocational training period only

The distribution of doctors by specialty across the different proportions of postgraduation rural training time is summarised for both MABEL and UQMediCoS datasets (Fig. 1). Notable differences are UQMediCoS had a higher proportion of doctors with mostly rural training time (91–100%), whilst MABEL had a higher proportion of doctors with minimal rural training time (particularly the 1–10% and 11–20% rural training categories). Substantial differences were seen by specialty with general practice much more likely to be associated with a higher proportion of rural training time in MABEL (31–40% through to 91–100% rural training). The differences by specialty are less stark in UQMediCoS, which relates to the prevocational training period only, when doctors are still navigating towards specialty training selection.

**Fig. 2:**
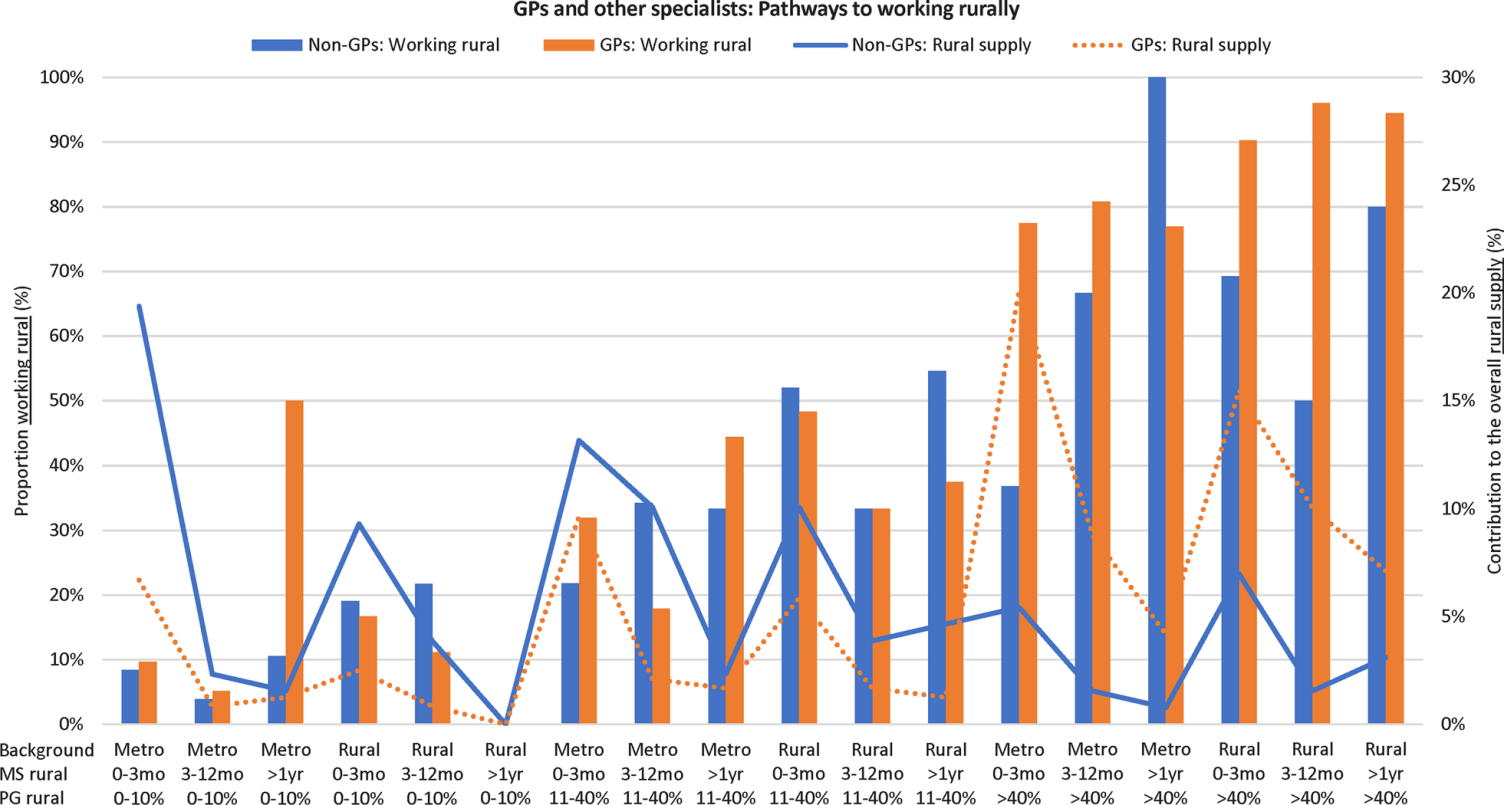
Contribution to the rural workforce per training pathway for MABEL’s consultant-level doctors (graduated 2000 +)

Table 4 confirms that most tested factors in the MABEL dataset were associated with training a larger proportion of their early career time in a rural area (stage 1 model). Moreover, the observed effect sizes (RRR, relative risk ratio) are substantially larger when associated with those spending above 40% of their postgraduation training time in a rural area. This includes rural background (RRR 2.9, 95% CI 2.1–4.2), > 1 year medical school rural (RRR 3.6, 95% CI 2.1–6.4), being rural bonded (RRR 3.6, 95% CI 2.1–6.1) and later choosing to work as a GP (RRR 7.0, 95% CI 4.7–10.4). Stage 2 modelling of current work location reveals the dominant impact of postgraduation rural time, with a very high odds ratio (OR) associated with rural practice for > 40% post-medical school time rurally for both GPs (OR 45, 95% CI 24–84) and other specialties (OR 11, 95% CI 5–22). For these models, being bonded and having a rural background remained significantly associated with working rurally, whereas medical school rural training time was no longer significant in the model.

**Table 4:** National (MABEL) graduates-logistic regression models of association between personal and pathway characteristics and training/practice outcomes

Factor	MABEL stage 1: outcome % rural training in first 10 years		MABEL stage 2: working rural (after fellowship)	
	11–40% (vs 0–10%)	> 40% (vs 0–10%)	General practice	Other specialty
	RRR (95% CI)	RRR (95% CI)	OR (95% CI)	OR (95% CI)
PG rural: (ref. 0–10%)	XX	XX	Ref	Ref
11–40% rural	XX	XX	3.59 (2.06–6.26)***	4.94 (3.01–8.08)***
> 40% rural	XX	XX	44.9 (24.1–83.5)***	10.7 (5.16–22.4)***
RurBG flag	1.55 (1.14–2.11)**	2.94 (2.05–4.22)***	2.16 (1.32–3.53)**	2.74 (1.70–4.42)***
MS rural (ref. 0–3 months)	Ref.	Ref.	Ref.	Ref.
3–12 months	1.58 (1.15–2.17)**	1.46 (0.97–2.18)	0.72 (0.42–1.27)	0.97 (0.56–1.67)
> 1 year	2.61 (1.57–4.32)***	3.62 (2.05–6.40)***	1.37 (0.62–3.03)	1.23 (0.58–2.63)
Bonded	2.49 (1.51–4.12)***	3.58 (2.11–6.07)***	3.14 (1.64–6.04)***	5.60 (2.36–13.3)***
Female	0.68 (0.51–0.90)**	0.73 (0.51–1.05)	0.94 (0.57–1.54)	0.95 (0.60–1.52)
Age 28 + at graduation	0.73 (0.53–1.01)	1.29 (0.89–1.86)	1.43 (0.88–2.23)	1.27 (0.73–2.19)
Specialty—GP	1.71 (1.29–2.27)***	7.01 (4.71–10.44)***	XX	XX

RRR = relative risk ratio; OR = odds ratio; RurBG = rural background; MS rural = medical school rural (clinical) training time; PG rural = postgraduation rural training time; GP = general practitioner

Table 5 identifies that no factors tested in the UQMediCoS dataset were associated with completing 11–40% prevocational training time in a rural area (stage 1), whereas rural background, completing 1 year or 2 years medical school rural training, being rural bonded (i.e. some rural return of service) and being male were all associated with completing more prevocational training time (> 40%) in a rural area (stage 1 model). Chosen specialty was not associated with prevocational time in a rural area. Stage 2 modelling revealed a significant association between > 40% prevocational rural training time and current rural practice, for both GPs (OR 3.8, 95% CI 1.6–9.1) and other specialties (OR 2.8, 95% CI 1.3–6.4). Medical school rural training remained significantly associated with rural practice amongst specialists other than GPs, whereas it wasn’t for GPs. Notably, rural background was also no longer significantly associated with rural practice amongst all doctors.

**Table 5:** UQ graduates-logistic regression models of association between personal and pathway characteristics and training/practice outcomes

Factor	UQ graduates: outcome % rural training in prevocational period		UQ graduates: working rural (vocational trainees and post-fellowship)	
	11–40% (vs 0–10%)	> 40% (vs 0–10%)	General practice	Other specialty
	RRR (95% CI)	RRR (95% CI)	OR (95% CI)	OR (95% CI)
PG rural: (ref. 0–10%)	XX	XX	Ref.	Ref.
11–40% rural	XX	XX	1.05 (0.32–3.47)	1.05 (0.38–2.93)
> 40% rural	XX	XX	3.80 (1.58–9.12)**	2.83 (1.25–6.39)*
RurBG flag	1.23 (0.65–2.31)	1.97 (1.15–3.39)*	1.97 (0.87–4.49)	1.60 (0.74–3.45)
MS rural (ref. 0 years)	Ref.	Ref.	Ref.	Ref.
1 year	0.56 (0.26–1.24)	2.51 (1.40–4.51)**	1.86 (0.64–5.42)	4.04 (1.84–8.89)***
2 years	1.39 (0.56–3.45)	6.28 (3.10–12.7)***	1.21 (0.40–3.68)	2.91 (1.08–7.81)*
Bonded	0.52 (0.17–1.55)	3.19 (1.69–6.03)***	2.66 (1.02–6.90)*	1.35 (0.51–3.59)
Female	0.73 (0.43–1.22)	0.58 (0.36–0.96)*	1.12 (0.52–2.42)	0.57 (0.28–1.16)
Age 28 + at graduation	0.57 (0.31–1.05)	0.74 (0.43–1.29)	2.66 (1.18–5.97)*	0.75 (0.32–1.75)
Specialty—GP	0.72 (0.40–1.27)	1.33 (0.80–2.21)	XX	XX

*RRR *relative risk ratio, *OR* odds ratio, *RurBG* rural background, *MS* rural medical school rural (clinical) training time, *PG rural* postgraduation rural training time, *GP* general practitioner

**Fig. 3:**
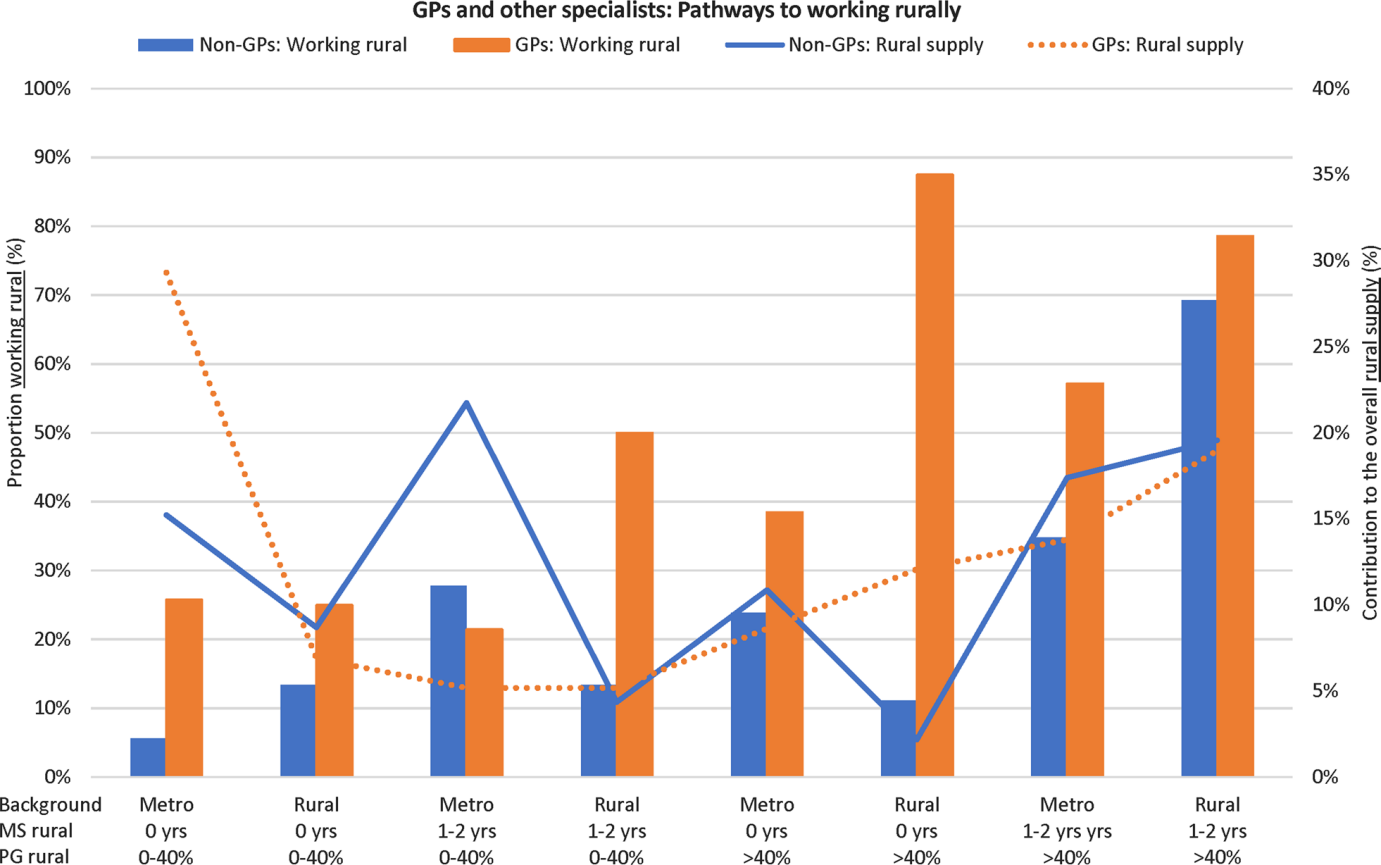
Contribution to the rural workforce per training pathway for UQMediCoS’s graduates (2002 + , excludes prevocationals)

Figures 2 and 3 visualise the key pathways to practising rurally for each of the MABEL and UQMediCoS datasets, for all combinations of rural background (2 levels), medical school rural training time (3 levels MABEL, 2 levels UQMediCoS) and postgraduation rural training time (3 levels MABEL, 2 levels UQMediCoS). In Fig. 2 (MABEL), there is a strong pattern of taller blue bars (higher % working rural) to the right for GPs, which consistently relates to pathways with > 40% postgraduation rural training time. In aggregate, these six pathways made up 66% of the supply of rural doctors (orange line). The pattern amongst other specialists is less stark, though there is clear growth of the blue line to the right of the graph. Additionally, those with > 40% postgraduation rural training time constitute only 19% of the ‘other specialty’ rural workforce. In Fig. 3 (UQMediCoS), those with > 40% postgraduation rural training time make up about 50% of the rural supply for both GPs and other specialists. The proportion working rurally is greater for all pathway combinations (rural background, medical school rural time, specialty) when comparing those with more prevocational rural training time (> 40% vs 0–40%).

## Discussion

**Fig. 4:**
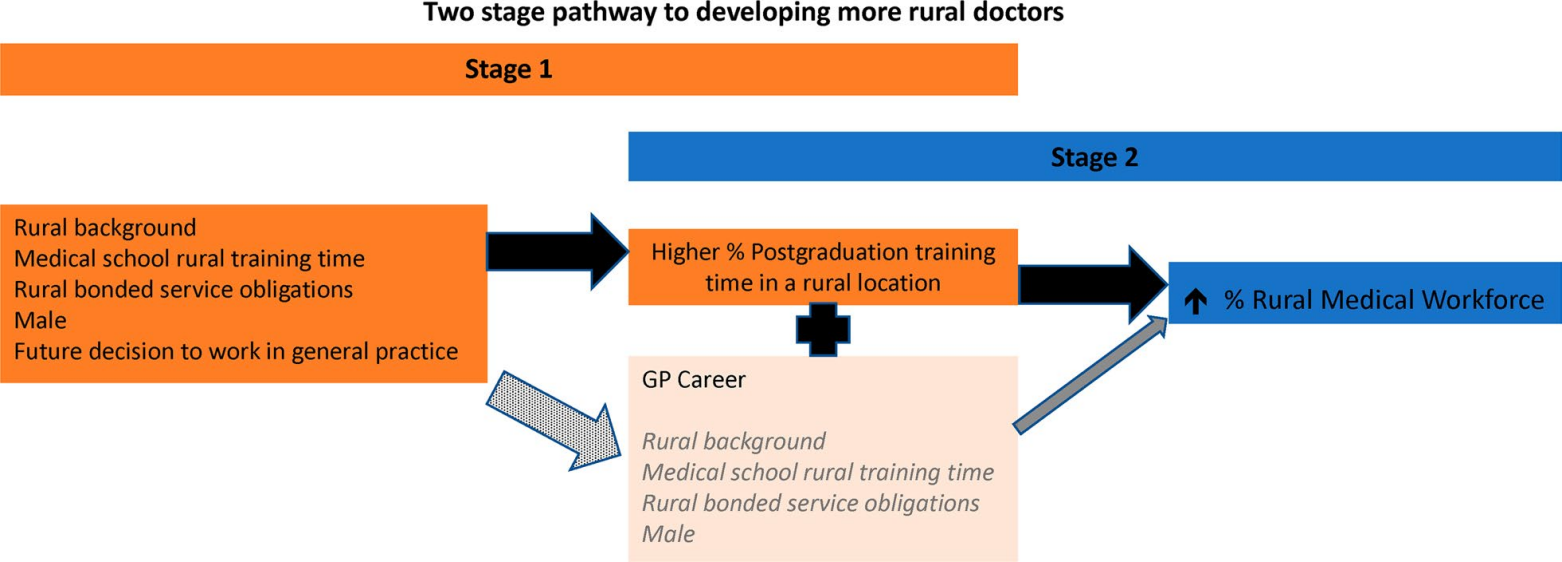
Summary of the role of ‘Higher % Postgraduation rural training time’ in both Stages 1 and 2 of the pathway to increasing the rural medical workforce

This study provides new evidence of the association between rural training time after leaving medical school and subsequent rural practice, after accounting for the contribution of the other key factors of childhood background and medical school rural training time across the entire training pathway. It reveals that distribution of the medical workforce is generally built upon in two stages, which is summarised in Fig. 4. In stage 1, commonly recognised factors like rural background, medical school rural training time, having rural bonded service obligations, future decision to work in general practice and being male were all significantly associated with spending a higher proportion of their postgraduation training time in a rural location. However, in stage 2 amongst early career consultants (MABEL dataset), it was seen that a higher proportion of postgraduation training in a rural location was by far the strongest factor relating to future practice in a rural location. The evidence was less overt in the UQMediCoS dataset (which aggregated registrars and consultants), though it too found a higher proportion of postgraduation rural training time was a significant factor, whereas rural background was no longer significant in stage 2 for each model, nor was medical school rural training time for GPs.

These results suggest that the training period shortly following medical school plays a substantial role in shaping the subsequent distribution of the medical workforce, for both GPs and other specialists. This finding is concerning given that many studies have identified barriers faced by junior doctors seeking specialty college entry that may be magnified (or perceived that way) when based outside of metropolitan locations [34–37]. Examples of this include poor recognition of rural training experience in specialty college selection criteria, experiences of stigmatisation relating to rural medicine, reduced access to research opportunities in rural areas, access to smaller networks of reputable consultants within their target specialty, as well as simply having fewer visible local pathways [15, 38, 39]. To date, most stakeholders responsible for prevocational and vocational training have lacked conviction in addressing these rural pathway shortcomings [2, 15]. A secondary concern from these results is that Australia has seen a large growth of wholly regional (end-to-end) medical school training, but this is generally not matched at the same rate with postgraduation regional training pathways. The results from the current study confirm that having one part of the pathway being strong but not matched by the other part will likely greatly dilute longer-term distributional outcomes.

Modelling the direct linkage between postgraduation rural training time and workforce distribution has infrequently been reported. This study highlights key differences of study outcomes depending on definitions used and observed training periods. MABEL data revealed a very strong association between postgraduation rural training time and current rural practice; however, this study's training time related to the first 10 years after medical school, constituting a large proportion of their educational pathway. In addition, the study’s inclusion criteria meant that observed work location relates to only a short period into their career as an independent consultant doctor, thus there may be some inertia effect observed whereby doctors remain working in the same (or similar) location as where their training was completed. The other contributor to the observed large effect size is the strong association between the reference group of 0–10% postgraduation rural training time and continuing to work in a metropolitan location (around 90%), thus magnifying the relative difference.

Previous studies of GPs completing their vocational training identified the strong contribution of recent rural training pathways to their current work location being rural [14, 17, 40], which this study confirms. It was similarly demonstrated that amongst nurses and allied health clinicians, having a rural job immediately after graduation was the key significant factor of longer-term rural practice [41]. These new findings are important in further understanding the contribution of various rural connection points towards a doctor choosing to work rurally. Childhood rural background and medical school rural training are the two most prominent factors widely demonstrated to be associated with an increased likelihood of practising in a rural location. This study clearly demonstrates these factors were critical influences of their choice to spend a higher proportion of their initial post-medical school training period in a rural area. However, the direct (measurable) impact of childhood and medical school rural connections may somewhat diminish once accounting for this postgraduation rural decision. This finding complements previous evidence from the UQMediCoS dataset revealing that a doctor’s certainty of wanting to practise rurally is most strongly developed within the early postgraduate training period [23].

The postgraduation training period relates to many doctors progressing from their mid-20s to their mid-30s, often characterised by key life stages like having children, solidifying life partner decisions, purchasing a home, and choosing where to ‘raise the family’. Many doctors have previously expressed the difficulty of medical training often requiring frequent moving houses between different training locations and difficulties satisfying both their professional and personal needs, particularly amongst female doctors in training [30, 42, 43]. Whilst this current study did not test retention in a specific region, these results appear to support that some locational stability may be desired in navigating these key life stages, once they decide which type of location they prefer to work in. Outside of general practice, such stability in this career stage is often difficult to achieve. One approach to address this may be having ‘deaneries’ similar to the United Kingdom, whereby a more localised regional-level responsibility for training pathways is utilised [44]. Australia’s relatively new national network of Regional Training Hubs [45], which are intentionally linked to the Rural Clinical Schools program in medical schools, is tasked with building training pathways within a region, though direct evidence of growth relating to this programme remains limited.

### Strengths and limitations

This study draws upon two large datasets with similar but not equivalent measures of different rural exposures. Differences of the postgraduation rural training time measurement necessitated analysis differences for each. MABEL measured postgraduation rural training time over 10 years, which is a substantial part of a doctor’s career. Moreover, it was not appropriate for the MABEL dataset to include data from registrars, thus both factors may somewhat artificially bloat the effect size for postgraduation rural training time, compared with the other factors. In contrast, UQMediCoS dataset’s smaller observation counts may be contributing to underpowered statistical models. Moreover, this prevented UQMediCoS from excluding data from registrars, which may lead to an underestimation of the effect size for postgraduation rural training time. This is due to the rotational nature of postgraduate training models meaning that the observed work location may not reflect their long-term outcome. Key independent factors of medical school rural time and postgraduation rural time may be subject to recall bias, with the exception of UQMediCoS utilising data linkage to their rural clinical training placements. Despite these limitations, this study is strengthened by combining and contrasting evidence from these two datasets.

## Conclusions

This study provides important new evidence of the significance of the rural training pathway beyond selecting more rural background students and training more students, for longer periods, in rural areas during medical school. It identifies that developing rural doctors aligns with two distinct career periods that are strongly linked. Firstly, the early stages (prior to completing medical school) are building their understanding of and interest in working and living rurally. Secondly, the later stages (postgraduation training) appear to be both their pathway to becoming a fully qualified doctor, but more importantly from a distributional perspective, confirming their longer-term decision to both work and live rurally. This evidence supports expansion of rural training pathways beyond medical school, aligning them with the needs of rural communities. Moreover, it encourages stakeholders involved in postgraduation training to be more cognisant of the impact of their decisions in supporting the growth of more rural-based opportunities can have on workforce distribution.

## Data Availability

The datasets used and/or analysed during the current study are available from the corresponding author on reasonable request.

## Abbreviations

GP: General Practitioner
MABEL: Medicine in Australia: Balancing Employment and Life (study)
OR: Odds ratio
RRR: Relative risk ratio
UQ: The University of Queensland
UQMediCoS: The University of Queensland’s Medical graduates Cohort Study
